# Supplementary material for: Are carbon emissions trading and green financial instruments synergistic? -Comprehensive quantitative research based on content analysis
Source: PLoS One. 2024 Mar 7;19(3):e0298601. doi: 10.1371/journal.pone.0298601 (PMC10919601; doi:10.1371/journal.pone.0298601)
Supplement: S1 Data — (ZIP) [file pone.0298601.s001.zip › Data/地级市Z_F工作报告.zip!/║╙─╧/╓▄┐┌╩╨/└┤╫╘╣1⁄2╓┌║┼í╛┬φ┐╦╩2╛▌═°í┐_2017.docx]

各位代表：

　　现在，我代表市人民政府，向大会报告工作，请予审议，并请各位政协委员和其他列席人士提出意见。

　　一、过去五年工作回顾

　　过去的五年，是周口发展面临重大挑战、取得显著成效的五年。五年来，在省委、省政府和市委的正确领导下，全市上下认真落实党的十八大和十八届三中、四中、五中、六中全会精神，深入贯彻习近平总书记系列重要讲话精神和调研指导河南工作时的重要讲话精神，迎难而上，砥砺前行，扎实做好稳增长、调结构、促转型、惠民生、防风险等各项工作，较好地完成了市三届人大历次会议确定的目标任务，不断开创了经济社会发展的新局面。

　　——这五年，是综合实力显著增强的五年。主要经济指标增速连续五年高于全省平均水平，生产总值由2011年的1407亿元增加到2016年的2260亿元，从全省第9位上升至第5位，年均增长9.3％，分别高于全国、全省2个百分点和0.44个百分点。一般公共预算收入103.9亿元，是2011年的2.1倍，年均增长16.4％。固定资产投资（不含农户）完成1862亿元，是2011年的2.5倍，年均增长20％。社会消费品零售总额突破1000亿元，年均增长13.5％。

　　——这五年，是产业结构不断优化的五年。三次产业结构由2011年的27.7︰46.5︰25.8调整优化为20.3︰45.9︰33.8，一产占比下降，三产占比增加，二三产业占GDP的比重达79.7％。一是工业支撑作用增强。规模以上工业企业新增330家，达1299家，增加值突破1000亿元，年均增长13.7%。阿尔本制衣年产120万套服装项目建成投产，鸿闽纺织产业集群初具规模，太康纺纱总规模超过150万锭，凯鸿、亚泰、费斯雅等36家制鞋企业落户周口，纺织服装（制鞋）产业规模达720亿元，鞋类出口占全省的29%。金丹乳酸生产规模亚洲第一、全球二是乐普、百年康鑫等药业发展势头良好，生物医药产业集群规模达792.1亿元。装备制造、电子信息、新型建材等产业集群不断壮大，主营业务收入分别达274.6亿元、370.2亿元、393.7亿元。太康锅炉、扶沟机械制造、西华无人机、沈丘聚酯网、淮阳塑料、项城和郸城医药等特色产业链条持续拉长，其中太康锅炉成为国家级区域品牌培育试点。建成省级以上企业技术中心28家、省级以上工程技术研究中心33家、市级以上工程技术中心127家、博士后科研工作站4家、院士工作站4家。荣获国家科技进步二等奖1项。培育驰名和著名商标73件及省长和市长质量奖23家，商标注册总量居全省第4位。产业集聚区建成区面积达106平方公里，完成固定资产投资1053.5亿元，入驻规模以上工业企业600家，吸纳就业31.1万人，5个被评为二星级、5个被评为一星级。二是农业综合效益提高。建成高标准农田633.7万亩，粮食综合生产能力稳步提升，周麦28、周麦30通过国家评审。我市被评为全国粮食生产先进市，8个县（市）被评为全国粮食生产先进县。种养结构持续优化，形成了林果蔬菜、花卉苗木、畜牧养殖等农业品牌。土地适度规模化经营稳步推进。培育农民合作社14155家，居全省第1位，其中国家级示范社39家。金丝猴集团与美国好时公司、普爱饲料与丹麦DLG集团实现战略合作，益海粮油、鲁花集团等产业化龙头企业不断壮大，食品加工产业主营业务收入达1298.6亿元，成为中国食品制造示范基地。建成省级农业产业化集群16个，实现销售收入750亿元。新增国家农业科技园1家、省级农业科技园3家。建成都市生态农业园区53个。发展标准化规模养殖场2475家，畜牧业产值1171.9亿元，8个县（市）被评为国家级生猪调出大县。完成林业生态工程50.6万亩。成功创建全国农业综合标准化示范市和全国平安农机示范市。三是服务业发展速度加快。第三产业增加值764亿元，是2011年的1.6倍，年均增长9.7%。全社会货运量突破9.2亿吨，其中港口吞吐量由121万吨增长到712万吨。服务业“两区”建设步伐加快，市商务中心区进入2016年度全省“十快”。华耀城商贸物流园区、项城欧蓓莎、郸城万洋国际博览城、太康万城金街、扶沟万鹤赉、沈丘玉文化产业园等一批商贸城项目建成投用。2家农信社组建农商行，6家村镇银行开业运营，86家企业在中原股权交易中心挂牌。阿里巴巴、京东商城、苏宁易购等知名电商入驻我市。淮阳获得国家级电子商务进农村示范县。旅游业接待总人数8572.1万人次，总收入实现396.9亿元。

　　——这五年，是城乡建设稳步提升的五年。完成公路投资143亿元，通车总里程1.9万公里，百平方公里路网密度提高17公里。周南高速开工建设。新周高速及周西、周淮、郸淮等快速公路建成通车，高速公路通车总里程496公里。新改建农村公路3400公里，改建桥梁1.9万延米。漯阜铁路升级为国家铁路，复线电气化改造全面完成，客车直达北京、上海、广州、天津等地。三洋铁路（周口段）顺利开工。郑合高铁（周口段）全线开工建设。航运建设完成投资22亿元，实现通航里程234公里。周口民用运输机场项目获得国家批复，周口西华通用机场加快建设，“公、铁、水、空”立体化大交通格局日益显现。2×66万千瓦燃煤电厂、2×45万千瓦燃气电厂建设步伐加快，电网建设投资64亿元，实现220千伏变电站县域全覆盖。管道天然气通达所有县（市）。实施农村安全饮水工程，解决395万农村居民和在校师生的安全饮水问题。开工建设棚户区改造安置房59319套，完成农村危房改造10.5万户。沙颍河城区东段治理基本完工，市县文化中心、体育中心、科技中心、新火车站、汽车客运总站、公园游园、城市综合体和“两小”市场等一批公共服务设施建成投用。建成南水北调东区水厂，中心城区部分区域和商水县城居民吃上了丹江水。城市污水处理率、垃圾无害化处理率分别提高到86%、89%。新型城镇化发展步伐加快，城镇化率达39.5%，提高8个百分点。实施整村推进项目1199个，建成市级产业扶贫示范基地121个，脱贫攻坚工作取得阶段性成效。中心城区成功创建省级文明城市，项城、沈丘保持省级文明县城称号，郸城、西华、淮阳创建为省级文明县城。12个乡镇成为全省美丽乡镇建设试点，1664个村成为农村人居环境达标村。

　　——这五年，是改革开放持续深化的五年。政府机构改革、公务用车制度改革全面完成。产业集聚区与乡镇行政区域管理套合稳步实施。“放管服”改革深入推进，取消、下放行政审批事项202项，政府权力清单、责任清单阶段性建设任务完成。县级公立医院综合改革覆盖率100％。商事制度改革步伐加快，市场主体26万户。财税、国企、交通、户籍、供销和国有林场等方面改革扎实推进。实际利用外资22.5亿美元，新增省外资金项目943个，实际到位省外资金1962亿元。正大、福喜、华耀城、富士康、碧桂园、娃哈哈、恒大等一批知名企业落户周口。外贸进出口完成36.1亿美元。成功创建河南省食品调味品出口基地、河南省纺织服饰出口基地。

　　——这五年，是社会民生明显改善的五年。农村劳动力转移就业新增54.3万人，城镇就业新增46.5万人。城镇居民、农村居民可支配收入分别达到22471元、9279元，年均增长9.6％、11.1％。居民存款余额1952.2亿元，是2011年的2.2倍。财政投入1586亿元，办成了一批民生实事。中小学薄弱学校改造加快推进，招聘教师2.3万人，32万余名考生升入高等学府，其中366名学子被清华、北大录取，打造了郸城一高等一批名校，创造了周口教育经验。新改建幼儿园1718所，学龄前儿童毛入园率85.2%，入园难、大班额问题得到缓解。新建一批职业院校，培养专业技能人才15.5万人次。新建村卫生室959个，新增卫生人员5205人、执业医师8869人，人均基本公共卫生服务经费提高20元。周口广电综合发射塔建成投用。新建文化活动室432个、农家书屋4284家。市戏剧艺术研究院连续5届荣获省戏剧大赛金奖，《天职》等一批剧目荣获国家级大奖。成功举办市第二届和第三届运动会、省第十二届老年人体育健身大会、周口马拉松暨中国慈善马拉松赛，获得省第十三届运动会承办权。发放资金45.6亿元，救助城乡低保对象300万人次。建成各类养老机构213个，专业养老床位3.2万张。城乡居民基本医疗保险实现整合并轨。企业退休人员养老金连年上调，养老金按时足额发放率和社会化发放率100%。环境质量持续改善，2016年成为全省唯一空气质量三项指标完成省定目标的省辖市。加强安全生产和食品药品监管，创新“一会四联”工作机制，深化平安周口创建，群众安全感和满意度不断提升。

　　五年来，市人民政府按照市委的部署，始终加强自身建设，认真开展党的群众路线教育实践活动、“三严三实”专题教育和“两学一做”学习教育，保持正确的政治方向。始终坚持依法行政，自觉接受人大法律监督、政协民主监督、社会舆论监督，认真办理人大代表建议和政协委员提案，支持民主党派、工商联和无党派人士参政议政。始终加强行政监察和审计监督，从严查处和纠正不正之风。五年来，国防动员和双拥工作健康开展，沈丘荣获全国双拥模范县称号。妇女、儿童、残疾人、红十字、慈善等事业健康发展，统计、外事、侨务、盐业、物价、海关、史志、档案、气象、地震、民族宗教、社会科学、人民防空、应急救援、对口援疆等各项工作均取得新的成绩。

　　各位代表！

　　回顾过去的五年，勤劳善良、自强不息的千万周口儿女，在这片充满希望的热土上，创造了无愧于时代的辉煌业绩！可以说，这五年，既是全市上下抢抓机遇、积极作为、开拓进取、务实重干的五年，也是经济社会发展较快、城乡面貌变化较大、人民群众获益较多的五年。这些成绩的取得，是省委、省政府和市委正确领导的结果，是人大、政协和社会各界监督支持的结果，是全市广大干部群众苦干实干的结果。在此，我代表市人民政府，向奋战在全市各行各业的广大劳动者，向市人大代表、政协委员，向各民主党派、工商联和无党派人士、各人民团体和离退休老同志，向中央、省驻周单位的同志们以及驻周部队指战员、武警官兵、政法干警，向所有关心、支持、参与周口建设和发展的朋友们，表示衷心的感谢和崇高的敬意！

　　在充分肯定成绩的同时，我们也清醒地看到，我们的工作与省委、省政府的要求和人民群众的期待还有一定距离，发展中还存在不少困难和问题。同时，我们还深刻认识到，问题的实质在于供需错配，产品的供给跟不上需求、资本的供给跟不上需求、项目的供给跟不上需求、环境的供给跟不上需求、作风的供给跟不上需求。这种严重的供需错配，导致加快发展的旧动能释放不出来、新动能培育不出来。一是产业结构不优，动能受到束缚。一产大而不强，虽然我市农业资源丰富，但多是低端、初级产品，高品质、高附加值的农产品供给不足；二产散而弱小，企业少、规模小，产业链条短、产品附加值低，企业核心竞争力弱，还处在价值链的低端；三产小而不活，没有摆脱传统服务业的发展模式，现代服务业比重低，临港经济尚未起步，信息经济、分享经济、创意经济等新经济培育不足。二是要素供应不上，动能“先天不足”。从资本看，银行存贷比严重失衡，资本外流突出，外资引进困难；从科技看，研发投入少，高端创新平台少，创新型企业少；从人才看，人才的引进吸附能力弱，缺乏中高端人才。三是中心城区落后，动能集聚度低。中心城区建成区面积较小，规划建设管理水平不高，缺乏产业特别是现代服务业的支撑，对资本、科技、人才等要素吸附能力弱，辐射带动力不强。四是项目质量不高，动能不够有力。一些项目投资强度低，建设周期长，对税收的贡献率不高；部分重点项目进展缓慢，甚至停工搁浅；招商引资的合同履约率、资金到位率、项目开工率有待提升，项目建设对经济增长的支撑力度不够。五是干部作风不硬，动能缺乏保障。个别干部缺乏担当精神和责任意识，干工作推推动动、不推不动，甚至推而不动，不同程度地存在“懒政怠政”“中梗阻”“假作为”“虚作为”等现象，一些领域腐败问题时有发生。

　　对于这些问题，我们不回避、不遮掩、不推卸。在各位代表和委员的监督支持下，一定扑下身子，强化工作措施，狠抓工作落实，推动问题解决，让发展的动能充分释放、竞相迸发，使发展的前景更加广阔、更加美好，绝不辜负人民的期望和重托。

　　二、今后五年的总体要求和目标任务

　　各位代表！

　　今后五年，既是全面建成小康社会的决胜期，也是全面深化改革的攻坚期，更是加快周口发展的关键期。综合分析，当前和今后一个时期，挑战与机遇并存，机遇大于挑战，我们仍处在可以大有作为的重要战略机遇期。要看到，当前，世界经济深度调整，不稳定性、不确定性因素较多，整体复苏艰难曲折；全国全省经济正处于增长动力转换的关键时期，经济下行压力依然较大，稳增长任务艰巨；我市基础差、底子薄，转型发展攻坚、农村脱贫攻坚、环境治理攻坚任务繁重，加快发展的压力较大。同时也要看到，我们的发展正面临着难得机遇，国家制定了“一带一路”战略，我省集粮食生产核心区、中原经济区、郑州航空港经济综合实验区、河南自贸区等国家战略于一身；省委、省政府着力发挥优势打好“四张牌”，把我市列入百城建设提质工程首批试点范围，这些重大机遇必将为周口发展带来更多的利好政策。更要看到，我们的发展也具有很多优势，如：人力资源、土地资源、农产品资源丰富；工业去产能、去库存压力小，消费市场广阔，通江达海的水运优势突出，尤其是随着郑合高铁、周口港的运营和机场的投用，临港经济、临铁经济、临空经济的发展将会产生叠加效应；地处中原城市群核心发展区，是郑州至合肥发展轴的重要节点、承接产业转移的示范区，可以说发展的机会更多、空间更大。尤其要看到，当前全市广大干部群众，思发展、谋发展、促发展的心情更加迫切，比干劲、比贡献、比速度的热情更加高涨，干事创业的浓厚氛围已经形成，只要我们乘势而上、凝心聚力，攻坚克难、奋力拼搏，周口发展一定能够创造新的辉煌！

　　按照市四次党代会的部署，结合“十三五”发展规划纲要，今后五年政府工作的总体要求是：全面贯彻党的十八大和十八届三中、四中、五中、六中全会以及即将召开的党的十九大精神，深入贯彻习近平总书记系列重要讲话精神和党中央治国理政新理念新思想新战略，按照“五位一体”总体布局和“四个全面”战略布局，认真落实省委、省政府和市委决策部署，积极融入全省实施国家战略的进程，坚持稳中求进工作总基调，坚持“一中心四组团五支撑”的发展格局，以推进供给侧结构性改革为主线，抢抓政策机遇、推动要素集聚，加快动能培育、释放经济活力，务实重干、敢于担当，加压奋进、跨越发展，确保如期全面建成小康社会，使周口人民有更强的获得感、幸福感、尊严感。

　　根据这一要求，今后五年全市经济社会发展的目标任务是：

　　——发展速度持续加快，综合实力更加强大。经济发展增速高于全省平均水平，财政收入增速高于全省平均水平，居民收入增速高于全省平均水平。全市生产总值和城乡居民收入比2011年翻一番以上，生产总值突破3500亿元，规模以上工业增加值突破1500亿元，一般公共预算收入突破160亿元。

　　——转型升级提质增效，产业结构更加优化。加快转型攻坚，突出发展质量和效益，二三产业比重每年提高1.5个百分点左右。强化工业实体。新增规模以上工业企业500家，高新技术企业达到50家，培育3个主营业务收入超100亿元和10个超50亿元的龙头企业，打造食品加工、纺织服装、生物医药3个千亿级产业集群和装备制造、电子信息、新型建材3个500亿级产业集群。优化农业供给。积极推进供给侧结构性改革，大力发展观光农业、旅游农业、生态农业等新业态，着力提高农业规模化、标准化、产业化水平，打造农产品高端供给。建成市级以上龙头企业260家、产业化集群50个，注册家庭农场8000家，培育国家级农业高新技术产业示范区1家、省级农业科技园区2家、市级农业科技示范园区10家。提升服务业水平。加快服务业“两区”建设，培育一批金融、物流、信息、文化、旅游、健康等现代服务业，服务业产值1400亿元。加快发展临港产业。支持淮阳龙湖国家湿地公园和太昊陵创建5A级景区、西华盘古女娲创世文化园创建4A级景区。

　　——基础设施逐步完善，城乡发展更加协调。以全国文明城市提名城创建为总引领，以创建省级卫生城、国家园林城、全国双拥模范城为抓手，完善基础设施，着力构建中心城区、县城、小城镇和新农村协调发展的现代城乡体系，城镇化率每年提高2个百分点左右，逐步缩小与全国全省的差距。城镇建设更有品质。高标准建设城市基础设施和公共服务设施，加快建设智慧城市和海绵城市，提高城市综合承载能力。集中精力加快中心城区建设，完成新一轮行政区划编制调整，拓展城市发展空间，实现建成区面积110平方公里、人口规模110万以上。建成引黄调蓄工程和中心城区水系治理系列项目，打通市区所有断头路，建成高铁片区，扮靓精品片区，全面提升城市的凝聚力、辐射力。建成30万人以上的县城5个、建成区3万人以上的中心镇6个。城乡交通更为便捷。建成周南、阳新高速公路周口段，实现“县县通高速”、中心城区与组团城市之间通快速公路，国省干线公路里程2215公里，农村公路里程2万公里。郑合高铁、三洋铁路周口段建成运营，争取洛阳—平顶山—周口—商丘城际铁路开工建设。完成沙颍河周口至省界航道升级改造工程，实现四级航道160公里常年通航，新增港口泊位43个。周口民用运输机场开工建设，周口西华通用机场建成投用，通用航空产业基地建设加快，立体化交通格局基本形成。能源供应更加充裕。推进电网建设和农村电网改造，燃煤、燃气电厂建成发电，打造全省先进生物质能示范基地。实施天然气通乡镇工程，1/3以上的乡镇用上清洁能源。

　　——环境治理日益强化，生态质量更加优良。实施蓝天、碧水、乡村清洁“三大工程”和大气、水体、土壤三大污染防治攻坚，各项指数持续优于全省平均水平，让周口的天更蓝、地更绿、水更清。坚决打好蓝天保卫战，空气优良天数保持稳定。全面消除中心城区和各县城黑臭水体，重要河流水功能区达标率80%以上，城市饮用水水源地水质达标率100%。农村生活垃圾和面源土壤污染得到有效治理，畜禽粪便基本实现资源化利用，危险废物无害化利用处置率95%以上。加快推进国土绿化，完成造林45.8万亩，城市建成区绿地率36%。清洁低碳技术广泛应用，节能减排等环境约束性指标达到省定目标。

　　——民生福祉不断改善，社会大局更加和谐。坚决打赢脱贫攻坚战，确保2018年实现农村贫困户脱贫、贫困县全部摘帽。持续加大投入，坚持每年办好“十件民生实事”。大力推进大众创业、万众创新，实现城镇新增就业35万人以上，登记失业率控制在4%以内。办好人民满意的教育，入托难、上学难、大班额等问题进一步得到缓解，幼儿园毛入园率90%以上，各县（市、区）通过国家义务教育发展基本均衡达标验收，高中阶段教育普及，新改建在校生不低于3000人以上的职业教育中心4个。继续实施中心城区教育振兴计划，全面提升中心城区教育质量。推进健康周口建设，健全分级诊疗机制，完善公共卫生服务体系，人均基本公共卫生服务经费标准100元以上，实现每万名居民有2—3名全科医生，每千名老人有35张专业养老床位。持续实施文化惠民工程，加快发展文化产业。推进体育事业发展，开展全民健身运动，办好省第十三届运动会。加快建设覆盖城乡居民的社会保障体系，基本实现法定人员全覆盖。创新社会治理，深化平安建设，筑牢安全防线，维护社会大局和谐稳定。

　　各位代表！

　　宏伟蓝图令人鼓舞，美好前景催人奋进。展望今后的五年，我们充满信心。只要我们牢牢把握这一轮重要战略机遇期，抢抓主动权，抢占制高点，团结拼搏、奋发有为，就一定能催生强大动能，推动经济社会持续快速发展，实现我们的宏伟目标！

　　三、2017年重点工作

　　2017年是新一届政府的开局之年，做好今年的工作非常重要。今年发展的主要目标是：生产总值增长8％以上，一般公共预算收入增长8%，全社会固定资产投资增长12％，社会消费品零售总额增长11％，进出口保持稳定，实际吸收外资规模与上年持平，实际到位省外资金增长7％，城乡居民收入与地方生产总值同步增长，城镇新增就业6.3万人以上，居民消费价格涨幅控制在3%左右，节能减排、环境保护等约束性指标完成省定目标。

　　完成上述目标，需要全市上下的共同努力。目前，时间基本过半。从一季度来看，经济增速低于全省0.1个百分点，规模以上工业增加值增速从全省第1位下滑至第10位。从4、5月份的经济运行情况看，下行压力依然较大，形势不容乐观，完成全年的目标任务很艰巨。我们必须抢机遇、抢时间、抢进度，把各项工作往前赶，重点是着力推进“四大攻坚”、奋力实现“四大突破”。

　　“四大攻坚”，即：转型发展攻坚、农村脱贫攻坚、环境治理攻坚、深化改革攻坚。

　　（一）着力推进转型发展攻坚。坚持把动能作为产业转型发展的核心，改造提升旧动能，培育激发新动能，新旧动能共同发力，为产业转型发展提供强有力的引擎。

　　突出工业支柱，厚植动能。工业是国民经济的主导产业。在推动产业转型发展攻坚中，坚持把工业当主力、打头阵、挑重担，积极推进“一去三改”，加快工业转型升级。一是壮大企业规模。积极改造传统产业，支持纺织服装、装备制造等产业拉长链条。重点培育关联度大、主业突出、创新能力强、带动性强的龙头企业，促进骨干企业做大做强。实施中小企业培育计划和产业提升计划，滚动实施100个技术改造专项，引导中小企业向“专精特新”方向发展。实施创新龙头企业和“科技小巨人”企业培育工程，壮大高新技术企业群体。启动市级重大科技专项，建设市级工程技术研究中心10家以上，培育高新技术企业3—5家。建立企业资源储备库，选择重点培育对象，一对一进行培育，力争培育一批高成长性企业。瞄准战略性新兴产业发展方向，加快培育生物医药、节能环保等产业，培育新的经济增长点。二是加快产业集聚。围绕六大主导产业，绘制产业图谱，实施“百千亿”优势产业集群培育工程，力争市级培育2个主营业务收入超千亿的产业集群，县级培育一批超百亿的产业集群。分类处置产业集聚区停产半停产企业和停建缓建项目，盘活厂房、设备、土地等资源要素。积极争星晋位，在“十强十快”和三星级产业集聚区上力争实现新突破。支持经济开发区争创国家级开发区，加快川汇区高新技术产业开发区发展。三是推进智能升级。抢抓国务院推进《中国制造2025》试点示范的机遇，积极推进制造业与“互联网 ”融合发展，着力发展智能制造，建设智能工厂，推进生产过程智能化，积极研发智能装备和智能产品。发挥我市中小企业多的优势，支持大中小企业融通发展，加快发展网络化协调研发制造、大规模个性化定制、服务型制造等新模式。四是加强品牌培育。大力弘扬“工匠”精神，引导企业围绕市场需求，增品种、提品质、创品牌，提高产品档次和质量。支持企业争获中国质量奖、省长质量奖、市长质量奖，争创名牌产品、驰名商标和著名商标，提升企业的核心竞争力。五是搞好服务保障。深化“政策落实进万企”活动，搞好产销、银企、用工、产学研对接，构建企业服务长效机制，着力解决制约企业发展的突出问题。弘扬企业家精神，加强企业家队伍建设，组织好企业家活动，努力打造一支高素质的企业家队伍。

　　提升农业供给，释放动能。加快推进供给侧结构性改革，优化农产品供给，提高综合效益和竞争力，加快动能释放。一是提高供给品质。进一步调优种养结构，大力发展优质小麦、优质花生、优质果蔬、优质草畜，扩大生猪、肉羊、肉牛等畜产品生产基地规模。以西华、扶沟为试点，启动农产品质量安全可追溯平台建设，提升农产品质量安全水平，增加农产品高端供给。二是增强供给效益。鼓励龙头企业通过订单生产、土地股份合作、土地托管等方式，形成“龙头企业 合作社 农户”模式，带动农民脱贫致富。依托五得利、大用、牧原等龙头企业，大力发展农产品和畜产品精深加工，建成农业产业化集群42个，培育一批农业品牌，把周口的农产品资源优势转化为经济发展优势。三是打造供给平台。强化科技引领作用，积极创建国家级农业高新技术产业开发区，支持扶沟创建国家现代农业产业园。突出抓好东新区建业绿色基地、商水万亩高效农业示范区、太康现代生态循环农业基地、沈丘厚德生态农业园、淮阳新陆春天农业观光园等示范园区发展，创建现代农业示范基地50个，建成市级农业科技园区5—7家。积极引进农业产业化集群龙头企业，每个县（市、区）至少引进1个投资5亿元以上的农产品加工项目。

　　强化三产拉动，激发动能。积极推动三产的新产品、新服务、新业态、新模式，形成新的增长点，激发更多动能。一是商贸物流业再加速。加快推进龙都红星广场、周口游乐文化产业园、恒大微购商城、昊宇商业综合体、豫东电子商务产业园区等服务业项目，力争固定资产投资200亿元、主营业务收入80亿元、税收5亿元。发挥港口优势，加快引进培育高附加值的先进制造、现代物流、研发服务、出口加工、教育培训等临港产业，重点培育大件物流、集装箱物流、综合保税物流。实施“互联网＋流通”工程，规划发展冷链物流，加快推进黄淮农产品物流中心、华耀城商贸物流园区等提档升级。二是现代金融业再加速。积极对接中央和省金融机构，引进银行、保险、证券等机构落户周口，壮大金融业规模。完成农信社改制组建农商行工作。设立创业投资基金和产业投资基金，促进大众创业、万众创新，推动产业转型升级。支持企业上市挂牌，积极推进金丹乳酸、凯旺电子上市。三是新兴服务业再加速。立足我市产业基础，积极培育软件开发、工业设计、信息服务、检验检测、服务外包等生产性服务业，延长工业产业链条。以教育园区、文化艺术中心、全民体育活动中心、健康养老中心为重点，谋划建设一批生活性服务业项目。以知识、技术、信息、数据等新要素为支撑，孵化培育科技型中小微企业，推动数字经济、分享经济、绿色经济等新经济发展。四是文化旅游业再加速。依托“伏羲、女娲、漕运”等特色文化，发挥“书法、美术、戏曲”等文艺群体作用，打造一批具有鲜明特色的文化创意产品和文化创意产业园。加快周口杂技文化产业园建设。研究制定全域旅游发展规划，推进民宿产业，抓好特色小镇，发展休闲度假、文化养生等深度旅游新业态，促进旅游业向全域旅游提升，培育增长新动能。

　　（二）着力推进农村脱贫攻坚。坚持把脱贫攻坚作为头等大事和第一民生工程，全力以赴打赢攻坚战，确保按时完成13.34万人的脱贫任务。

　　突出产业扶贫。在做好问题整改、规范建档的基础上，把产业扶贫作为治本之策，常抓不懈、毫不放松。依托“种植、养殖、加工、光伏”等业态，建成100个高标准精准扶贫示范基地，带动贫困户持续增收。大力推广“巧媳妇”工程，创建456个标准化“扶贫车间”，让贫困群众在家门口就业。继续搞好与银行、保险机构合作，完善市县两级统贷平台，争取更多的金融资本支持扶贫开发。

　　完善基础设施。对照贫困县脱贫摘帽和全面小康标准，统筹整合涉农资金，实施专项行动计划，着力在村活动室、安全饮水、道路硬化、文化广场、卫生室建设等方面补短板。着力解决88.4万人的安全饮水问题，改建农村公路2100公里、改造农村危房5400户，完成7个县、300个村的电网改造任务，实现388个贫困村通动力电。统筹贫困村、非贫困村基础设施建设，做到统一规划、协调推进。

　　落实帮扶责任。严格落实市县主体责任、部门协同责任、乡村包干责任、驻村帮扶责任、督查巡查责任，强化驻村工作帮扶、非贫困村分包帮扶、扶贫日入户帮扶，不折不扣落实对贫困户的医疗、教育、危房改造等扶持政策。坚持“扶贫先扶智”“扶贫先扶技”，加强对贫困群众自主创业、转移就业能力的培训。宣传一批自强不息、勤劳致富的好典型，激发贫困户脱贫的内生动力。严守法纪红线，对作风不实、弄虚作假、截留挪用扶贫资金等行为，一律严肃处理。

　　（三）着力推进环境治理攻坚。紧盯PM10不高于101微克/立方米、PM2.5不高于61微克/立方米、空气优良天数不低于210天的目标，坚持源头严防、过程严管、后果严惩，努力完成攻坚任务。

　　加大防治力度。一是坚决遏制大气污染。完成电代煤、气代煤合计6万户和55台10蒸吨及以下燃煤锅炉拆改工作。全面开展对扬尘污染、各类油烟污染、秸秆和垃圾焚烧等方面的专项治理，加快淘汰黄标车，关闭处理小散乱污企业，推进臭氧污染治理，坚决打好蓝天保卫战。二是深入治理水体污染。实施碧水清洁工程，推进雨污分流和截污纳管，促进城区黑臭水体治理。加强饮用水水源地保护，抓好重点流域、重点领域的水污染防治，提高河道断面达标率。全面推行河长制，健全河湖水系管护长效机制。三是加快规范垃圾处理。深入开展清洁行动，新增城市建筑垃圾资源化利用能力100万吨以上，新增处理畜禽粪便能力8万吨/年。完成禁养区规模养殖场关闭搬迁工作。各县（市、区）列出专项经费，按行政村人口2％。的标准在村里配备保洁员，9月底前全面完成农村陈旧垃圾清理和集中堆放任务。有条件的地方，鼓励政府购买服务，积极探索推行“村收集、乡转运、县处理”模式，切实解决垃圾乱堆乱放无人管的问题，完成省定农村人居环境建设考核目标任务。

　　加强生态建设。全面加强水流域河道治理，完善两岸绿地、游园建设。积极申报国储林项目，实施森林城市、森林通道、森林社区、森林田园、森林乡村“五大工程”，提高城区公园绿地面积，新增造林绿化面积7.2万亩。

　　落实监管责任。坚持“党政同责、一岗双责、失职追责”，严格落实生态环境保护责任。严把环境准入关口，从源头上防止环境污染和生态破坏行为。实施节能减排降碳行动计划，完成省定减排任务。建立和完善生态补偿机制。严格耕地保护，确保耕地红线。健全联合执法体系建设，加大环境执法监察力度，严厉打击环境违法行为。

　　（四）着力推进深化改革攻坚。改革是释放红利、激发动能的重要举措。政府抓改革，核心是处理好政府与市场的关系，重点是深化各类经济体制改革，为持续健康增长增强动能。

　　深化国有企业改革。认真落实省委、省政府的部署和要求，扎实推进国有企业改革，促进国有资产保值增值。一方面，突出产权结构改革。产权是国企改革的核心和关键。通过资产重组、债务重组、产业重组等方式，推进国有企业产权多元化，组建一批能够有效参与市场竞争的企业集团。年内完成市保安服务公司、水利勘测设计院等企业的改制任务。各县（市、区）结合实际，完成一批公司制股份制改革任务。另一方面，推进治理结构改革。在坚持和加强党的领导的前提下，按照“产权明晰、权责明确、政企分开、管理科学”的现代企业制度要求，理顺董事会、监事会、经理层之间的关系，推行内部三项制度改革，实现用工、人事和分配与市场接轨。建立健全权力清单、责任清单等制度，使国有企业内部运行更加规范、高效。做好国有企业职工家属区“三供一业”剥离移交工作，全面完成省属企业离退休职工社会化管理移交工作。

　　深化“放管服”改革。按照国务院和省政府的新要求，在“放”上下功夫，在“管”上做文章，在“服”上促创新，加快减权、规范用权、查处滥权，做到审批更简、监管更强、服务更优。进一步推动“证照分离”，实施“多证合一”，为促进就业创业降门槛；进一步落实减税降费政策，为各类市场主体减负担；进一步梳理审批事项，建立审批清单，优化审批程序，简化审批手续，压减工业产品生产许可，为激发有效投资拓空间；进一步加强事中事后监管，推动“双随机、一公开”监管全覆盖，为公平营商创条件；进一步深化政务公开，实现政务云平台与省平台对接，推行市县乡三级“一号一窗一网”服务，为群众办事生活增便利。

　　完善新型城镇化体制机制。持续深化户籍制度改革，扩大户籍业务网上办理范围。加快构建以居住证制度为载体的基本公共服务供给机制。完善推进非户籍人口落户的政策措施。加快公租房建设，提高分配入住效率。加快推进农村土地承包经营权确权登记颁证工作。认真落实农民承包土地所有权、承包权、经营权分置政策，健全土地流转市场，稳妥推进农村宅基地制度改革等试点，促进新型城镇化进程。同时，进一步深化财税体制改革，加强债务管理，防范和化解债务风险。加快推进医药卫生体制、盐业体制、供销社、国有林场等改革。

　　“四大突破”，即：城市规划与建设管理实现突破、开放招商与项目建设实现突破、棚户区改造与“两违”治理实现突破、社会事业与民生改善实现突破。

　　（一）城市规划与建设管理实现突破。城市的规划建设管理水平，体现了一个城市的内在品质和外在风貌。中心城区的相对落后，集中体现在规划建设管理水平的落后上。改变城市面貌，提升城市形象，必须在规划、建设和管理上下功夫。

　　坚持规划先行。进一步完善总体规划，高起点、高标准编制专项规划及城市控制性详细规划。对环城道路、高铁片区、出入市口等重点项目、重要路段、重要节点，着力编制好修建性详细规划。对城市天际线进行总体设计，对干道红线、绿地绿线、河道蓝线、文化紫线等进行控制，对行道树、路沿石、人行道地砖等细节部分精心选择、精心设计、精心施工，保证城市整体风貌。坚决维护规划的严肃性，严格按照规划进行建设管理，对违反规划的建筑，坚决处置，确保规划的权威。

　　加快推进建设。抓住百城建设提质工程机遇，加快城市基础设施项目建设，提升公共服务供给水平。加快建设周口大道以东、周项路以北、太清路以南、武盛大道以西约35平方公里的精品片区。启动高铁片区建设，开工建设彩虹大道、淮阳路、平安路等地下综合管廊工程。提升改造中心城区城市出入市口。加快建设八一路南通商水工程、周项快速公路、汉阳路沙颍河大闸桥拓宽工程。开工建设新北外环路、文昌大道东延、八一路北延、开元大道东西连接、大庆路北延至新北环等工程。加快实施高速公路周口港区、经济开发区互通立交以及周口北、周口东高速收费站改扩建工程。启动武盛大道南延、七一路东延、中原路等一批断头路打通工程。实施城市主干道、重点建筑亮化美化工程。建设一批社区公园、街头游园、公共绿地。

　　严格城市管理。加快城市执法体制改革，年底前完成改革任务。以省级卫生城市创建为抓手，以环境卫生和出店经营集中整治为开始，持续开展专项整治行动，重点治理三轮车和机动车乱停乱行、乱闯红灯，渣土车滴撒抛漏以及乱堆乱放、乱贴乱画、占道经营等行为。中心城区主次干道和县级城市主干道机械化清扫率100%，上足机械、人工和设施，实现全天候保洁。探索城市管理长效机制，加强精细化管理，促进管理更加科学。县城和16个重点镇，切实加强规划、建设和管理，提高综合承载能力。抓好“农村新型社区”遗留问题整改，完成省定责任目标。

　　在加强城市规划、建设、管理的过程中，突出在水的方面彰显特色、寻求突破，做足做好做活水文章。周口三川交汇、大河穿城，是目前全省唯一通江达海的省辖市，水的经济、历史、文化、生态价值十分突出，我们一定要做足、做好、做活水文章，让水为周口人民造福。加快推进城市水系规划，统筹考虑航运、防洪、景观等功能，对沙颍河两岸和上游生态湿地以及引黄调蓄工程周边的天际线、绿化、景观、夜景亮化等进行整体设计。开工建设7条水系治理工程和引黄调蓄工程。启动沙颍河老城区段两岸拆迁改造。加快谋划两岸亲水区域、上游湿地公园建设。着力打造富有周口特色的景观长廊，提升中心城区的宜居度、美誉度。

　　（二）开放招商与项目建设实现突破。把项目建设牢牢抓在手上，大上项目、上大项目，不断壮大动能。

　　扩大开放抓招商。抢抓北京疏解非首都功能、雄安新区产业转移和我省实施国家战略的机遇，积极对接全省百城建设提质工程等政策，以“招商引资深化年”和“对外开放提升年”为抓手，完善专项招商计划，力争签约一批投资强度大、税收贡献高的项目。跟踪重庆招商活动项目落地，筹备好北京、温州等专项招商活动，举办好2017中国临港经济发展论坛。加强与河南自贸区对接，启动申建周口港综合保税区、周口口岸，加快物流园区、中联盛公共保税中心建设。依托郑州跨境电子商务试验区，启动“跨境电商进周口”活动，力争实现50家以上企业开展跨境电商业务，线上交易额1亿美元以上。实际吸收外资争取实现5.2亿美元，引进省外资金485.2亿元，进出口完成51.8亿元。

　　强力推动抓进度。围绕33个省级重点项目、272个市级重点项目，尤其是中心城区“四个一批”项目，坚持重点项目建设协调推进周例会和领导分包制度，全面加快在建项目进度。再集中开工一批项目，确保完成年度投资计划。加快推进金丹乳酸年产1万吨丙交酯、乐普药业年产30亿粒固体剂生产、润泰纺织服饰、金丝猴食盐深加工、淮阳瑞能服帽生产、扶沟静脉产业园、西华杭州刺绣产业园等项目。加快推进上亿国际中心、摩天高新农业生态园、水之蓝国际商贸城等项目。全面开工沙颍河周口至省界航道升级开发和阳新高速公路（周口段）工程，加快推进东区污水处理厂、洺河综合整治等项目。

　　竭诚服务抓保障。扎实开展突出问题集中整治行动，盘活“批而未供”“供而未用”等存量资源，积极向上争取用地指标增量，提高用地效率，保障重点项目用地需求。加快周口豫资公司、市投资集团融资平台的运营，做好重点项目、重点区域周边土地收储工作，控制一级市场，撬动更多资本投入城市建设。用好PPP模式，充分发挥市级投融资平台作用，探索建立重点项目融资建设新模式，积极争取社会资本支持。坚持重拳出击，综合施策，开展项目周边环境专项整治，为项目建设提供更好的环境服务。

　　(三)棚户区改造与“两违”治理实现突破。目前，中心城区建成区约40%为棚户区，还有10万多户的改造任务，亟待加快推进。重点推进沙北滨河棚户区、贾鲁河东街坊、西城棚户区、东成棚户区、界牌街棚户区、关帝庙周边棚户区等区域改造，全面完成年度任务。突出抓好市民中心和高铁片区等重点区域城中村拆迁改造工作。积极用好住房公积金等政策性金融服务工具，提高棚户区货币化安置比例。围绕打造中高端城市综合体，加快推进原荷花市场片区整体改造升级。各县（市）按照年度计划，完成棚户区（城中村）改造工作。

　　依法加强“两违”治理。对新增“两违”零容忍，对存量“两违”痛下决心，重拳出击，依法依规进行拆除，坚决打击阻挠拆除违章的个人，对不作为的责任人严格问责、追责，在全市形成不敢违建、不想违建、不能违建的氛围。

　　（四）社会事业与民生改善实现突破。民心是最大的政治。人民政府必须坚持不懈为人民群众办实事、解难事，不断保障和改善人民群众的生活。

　　大力促进创业就业。实施大学生就业促进和创业引领计划，支持农民工返乡创业就业，做好退役军人安置和创业服务工作。扶持城镇困难人员、残疾人就业创业，确保零就业家庭至少有一人稳定就业。实现城镇新增就业6.3万人，城镇登记失业率控制在4%以内。

　　加强社会事业建设。优化学校布局，加快标准化学校建设，改善984所薄弱中小学办学条件，推动郸城、扶沟、商水通过国家义务教育均衡达标验收。支持科技职业学院升本、联营职专和幼师升专、高级技工学校晋升技师学院，支持职业技术学院加快发展，师范学院建设应用型高校。推进市中心医院新区医院、市中医院东区分院等重点项目建设，扩大优质医疗资源供给。实施食品药品安全放心工程，启动省级食品安全市创建工作。加大国家“学生饮用奶”推广力度，确保供奶质量。建成投用市科技馆二期，启动建设周口大剧院。大力推进社会主义核心价值观建设和群众性精神文明创建工作。加强农村、社区综合性文化服务中心建设，实施数字图书馆工程，组织好“送戏下乡”等文化惠民活动。

　　提升社会保障水平。加快实施社保征缴体制改革，依法扩大社会保险覆盖面。推进新农合与城镇居民医疗保险制度整合衔接。对建档立卡贫困人口、特困人员救助供养对象、城乡最低生活保障对象、困境儿童等困难群众，按照每人60元的财政补助标准，建立大病补充医疗保险制度。全面落实困难残疾人生活补贴和重度残疾人护理补贴月人均不低于60元的标准。城乡低保月人均财政补助标准分别由不低于240元、132元提高到不低于250元、142元。社会散居孤儿、机构供养孤儿养育标准由每人每月不低于600元、1000元提高到不低于700元、1100元。继续提高退休人员基本养老金，稳步提高优抚标准。

　　全面推进社会治理。深化平安周口建设，推进“七五”普法，加大矛盾纠纷排查调处力度，依法严厉打击各类违法犯罪行为。落实信访工作责任制，依法解决群众合理诉求。严格落实安全生产责任制，深化重点行业、重点领域专项整治，坚决遏制重特大事故发生。防范打击非法集资，坚决守住不发生区域性、系统性金融风险的底线。

　　这里需要特别说明的是，省第十三届运动会明年将在我市举办，这既是全市的一件喜事，更是一件大事。现在距省运会开幕时间紧、任务重，必须举全市之力，在场馆建设、接待保障、安全保卫、城市管理、开闭幕式策划等方面抓紧筹备、加快推进，全力以赴把十三届省运会办成一届精彩热烈、富有特色、彰显形象的体育盛会。

　　同时，继续开展双拥共建，积极争创省级双拥模范城。支持军队改革和驻周解放军、武警部队、预备役部队、民兵队伍建设，推进军民融合深度发展。深入推进人民防空改革发展。维护农民工、妇女、儿童、老人合法权益，关心慈善、残疾人事业。加强民族宗教工作，支持工会、共青团、妇联、工商联等人民团体在经济社会发展中发挥桥梁纽带作用。做好统计、审计、公积金、外事、侨务、物价、海关、气象、地震、史志、社会科学、应急救援等工作。

　　四、全面加强自身建设

　　各位代表！

　　人民政府的工作，从根本上说就是为人民服务、对人民负责、受人民监督、让人民满意。实现这一目标，就必须加强政府自身建设，全面提高为人民服务的能力和水平，努力打造一支忠诚、干净、担当、务实的政府干部队伍。

　　政治立场更坚定。深入推进“两学一做”学习教育，牢固树立政治意识、大局意识、核心意识、看齐意识，始终在思想上政治上行动上同以习近平同志为核心的党中央保持高度一致。认真贯彻省委、省政府决策部署，严守政治纪律，恪守政治规矩，坚决服从市委领导，坚决贯彻市委决策，坚决落实市委决定。始终坚持全心全意为人民服务，坚持以人民为中心的发展思想，倾听群众心声、反映群众意愿、解决群众困难。

　　思想观念更解放。坚持“跳出周口看周口”，以更大的气魄、更宽的视野、更高的眼界来谋划和推动工作。敢于向省内外先进地区看齐，学习好经验、好做法，勤于思考，大胆实践。善于看大局、谋大势，不争论、不折腾、不埋怨，把精力放在抓发展上，做到聚精会神、心无旁骛，以成果论英雄，看政绩比能力。坚决摒弃安于现状、不思进取的心态，做到敢想敢干、敢闯敢试，敢于创造性地开展工作。以“报民恩，当不遗余力”的精神，敢于涉险滩、勇于过暗礁、善于闯激流，做到事业至上、发展为重，不断推动各项工作提速、提质、提效。

　　工作作风更务实。必须敢担当，把该负的责任负起来，把该做的工作做到位，不回避矛盾，不推诿扯皮，不患得患失。建立容错纠错机制，为担当者担当、替负责者负责。必须讲执着，坚持“谁牵头、谁负责，谁主抓、谁协调”的原则，对所负责的工作紧盯不放、跟踪问效、一抓到底。必须重实干，强化“鼓起劲来求发展、弯下身来抓落实”的意识，不喊空的、不做假的、不吹大的，不搞形式主义，不搞花架子，不造虚假政绩，脚踏实地，埋头苦干，以等不得、慢不得、拖不得的紧迫感往前赶、争一流，树立“靠担当推动实干、靠实干取得业绩、靠业绩体现作为”的价值取向。

　　依法行政更坚决。全面实施政府法律顾问制度，深化服务型行政执法创建活动，筑牢法治理念，增强尊法学法守法用法意识。坚持把依法行政、依法办事的原则和要求贯穿于政府决策、执行的全过程，全力打造法治政府。充分听取各方面意见，自觉接受人大法律监督、政协民主监督、社会舆论监督，让权力在阳光下运行。

　　廉洁建设更严格。严格落实党风廉政建设责任制，认真履行“一岗双责”，切实加大反腐倡廉力度。加强行政监察和审计监督，驰而不息改进作风，厉行勤俭节约，严防“四风”反弹。政府干部必须遵守中央八项规定和省市关于廉洁建设的各项要求，心存敬畏、言有所规、行有所止，严守为官做人的底线，着力营造清朗政风，维护人民政府的良好形象。

　　各位代表！

　　人民寄予厚望，报答唯有实干。只有实干，才能实现愿景；只有实干，才能不负重托。周口的发展无过客，周口的建设无坐客，周口的事业无看客。让我们在省委、省政府和市委的坚强领导下，振奋精神、坚定信心，同心同德、务实重干，为决胜全面小康、实现跨越发展而努力奋斗！
